# Assessment of Image Quality Performance of a Photon-Counting Computed Tomography Scanner Approved for Whole-Body Clinical Applications

**DOI:** 10.3390/s25237338

**Published:** 2025-12-02

**Authors:** Francesca Saveria Maddaloni, Antonio Sarno, Alessandro Loria, Anna Piai, Cristina Lenardi, Antonio Esposito, Antonella del Vecchio

**Affiliations:** 1 Department of Physics ‘Aldo Pontremoli’, University of Milan, 20133 Milan, Italy; antonio.sarno@unimi.it (A.S.); cristina.lenardi@unimi.it (C.L.); 2 Milano Division, National Institute for Nuclear Physics, 20133 Milan, Italy; 3 Medical Phyisics Department, IRCCS San Raffaele Hospital, 20132 Milan, Italy; loria.alessandro@hsr.it (A.L.); piai.anna@hsr.it (A.P.); delvecchio.antonella@hsr.it (A.d.V.); 4 Experimental Imaging Center, Vita-Salute San Raffaele University, 20132 Milan, Italy; esposito.antonio@hsr.it

**Keywords:** photon-counting detectors, task-based metrics, CT image quality

## Abstract

Background: Photon-counting computed tomography (PCCT) represents a major technological advance in clinical CT imaging, offering superior spatial resolution, enhanced material discrimination, and potential radiation dose reduction compared to conventional energy-integrating detector systems. As the first clinically approved PCCT scanner becomes available, establishing a comprehensive characterization of its image quality is essential to understand its performance and clinical impact. Methods: Image quality was evaluated using a commercial quality assurance phantom with acquisition protocols typically used for three anatomical regions—head, abdomen/thorax, and inner ear—representing diverse clinical scenarios. Each region was scanned using both ultra-high-resolution (UHR, 120 × 0.2 mm slices) and conventional (144 × 0.4 mm slices) protocols. Conventional metrics, including signal-to-noise ratio (SNR), contrast-to-noise ratio (CNR), slice thickness accuracy, and uniformity, were assessed following international standards. Task-based analysis was also performed through target transfer function (TTF), noise power spectrum (NPS), and detectability index (d′) to evaluate diagnostic relevance. Results: UHR protocols provided markedly improved spatial resolution, particularly in the inner ear imaging, as confirmed by TTF analysis, though with increased noise and reduced low-contrast detectability in certain conditions. CT numbers showed linear correspondence with known attenuation coefficients across all protocols. Conclusions: This study establishes a detailed technical characterization of the first clinical PCCT scanner, demonstrating significant improvements in terms of spatial resolution and accuracy of the quantitative image analysis, while highlighting the need for noise–contrast optimization in high-resolution imaging.

## 1. Introduction

Computed tomography (CT) has become an indispensable tool in modern medical diagnostics, offering detailed anatomical visualization that supports disease detection, treatment planning, and patient management across a wide range of clinical fields, including oncology, cardiology, and radiotherapy. Since its introduction in the 1970s, CT technology has experienced several technical advancements, such as the introduction of multidetector, spiral scanning geometry and, more recently, dual-source scanners, and continues to increase our knowledge about diseases and the management of major health [1,2]. Despite these advances, conventional CT scanners equipped with energy-integrating detectors (EIDs) still have some limitations regarding electronic noise and spatial resolution, which could affect diagnostic performance in some applications [3]. Additionally, the energy dependence of the EIDs reduces the contribution of the low-energy photons to image generation, with these carrying most of the contrast signal. The introduction of photon-counting CT (PCCT) marks a paradigm shift in CT imaging. By employing photon-counting detectors (PCDs), PCCT systems permit the direct conversion of impinging photon energy into an electric signal (electron–hole pairs), allowing a reduction in thermal noise and the possibility of photon energy discrimination [4,5,6,7]. Each photon is individually counted whenever it produces an electrical signal that is higher than a calibrated threshold, excluding the electronic noise from the acquired projections [8]. The direct conversion wafer constituting the PCD—usually made of high-Z semiconductor materials such as cadmium telluride (CdTe) or cadmium zinc telluride (CZT) [9]—avoids the signal spreading that characterizes the light emission in scintillators embodied in EID-based computed tomography (EID-CT) and leads to drastic improvement of the image spatial resolution and the conspicuity of fine details [4,5]. Additionally, the use of multiple thresholds in the counting processes opens the possibility to implement dispersive algorithms to decompose the material and tissue concentrations within the reconstructed voxels [10,11,12], with the perspective of new paradigms in the field of CT imaging [9,13,14]. Although the use of PCD detectors in medicine has been investigated for more than two decades, recent innovations have permitted the technical characteristics of PCCT scanners to be compliant with the clinical demand. Hence, several challenges, mainly related to the frame rate and dimensions, had prevented the use of PCDs in substitution of EID. These shortcomings—that limited the accepted photon fluxes, detector frame rate, and dimensions of the PCD—had mainly been related to pile-up, charge sharing, polarization, and detector uniformity and stability [9]. In recent years, features related to spatial resolution and noise reduction have made such a technology a suitable tool for breast imaging, overcoming limitations in microcalcification visibility and doses of dedicated CT scanners based on EID flat panel detectors [15]. Hence, the PCCT scanner dedicated to breast developed by AB-CT [15] received a CE mark, and it is today largely investigated in clinical practice featuring a spatial resolution as high as 5 ${\mathrm{mm}}^{-1}$ (MTF at 10%) and average glandular dose to the irradiated breast with no statistical difference to that used in digital breast tomosynthesis [16]. However, the AB-CT scanner does not exploit several energy thresholds for material decomposition applications. On the other hand, the FDA-cleared Samsung NeuroLogica PCCT scanner dedicated to the brain imaging offers the possibility of distinguishing photon energy in three energy bins with the intent of estimating calcium and iodine maps in reconstructed images [17]. Among several vendors with PCCT scanner prototypes in advanced research stage [9], in today’s state-of-the-art, the Naeotom Alpha® produced by Siemens Healthineers (Erlangen, Germany) is the first whole-body clinical PCCT system to receive the FDA clearance. It may reach an ultra-high spatial resolution relying on the QuantaMax detector elements with pixels of 0.150 mm × 0.175 mm [18]. This whole-body scanner is provided with two X-ray tubes, allowing a better temporal resolution mainly for cardiac applications (66 ms). Another advancement in CT technology affecting also the PCCT is represented by the introduction of dedicated iterative reconstruction (IR) algorithms, which can replace filtered backprojection (FBP) methods [19,20,21]. The Naeotom Alpha PCCT scanner adopts both FBP and IR methods, and the non-linearity introduced by the latter is a challenging feature in the evaluation of technical image quality, requiring ad hoc approaches for image quality assessment and comparisons. Over the past few years, several studies have demonstrated the clinical potential of PCCT in areas including cardiac, pulmonary, spectral, and functional imaging [22,23,24,25,26,27,28]. However, only a few works [10,17,28,29,30,31] have quantitatively assessed technical image quality parameters in an FDA-cleared clinical PCCT scanner, and even fewer have provided a comprehensive, task-based analysis aligned with medical physics standards.

The present work addresses this gap by providing the first systematic and comprehensive technical characterization of an FDA-cleared clinical PCCT scanner, with a focus on standard adult imaging protocols. Using a dedicated phantom-based methodology to ensure the reproducibility of the measurements carried out, we evaluated both basic and task-based image quality metrics, quantifying their relationship with radiation dose and reconstruction technique. More in detail, as also described by the AAPM TG-233, the quasi-linear reconstruction methods provided by iterative algorithms make the task-based analysis necessary for this type of investigation [32]. This study offers new, reproducible benchmark data for PCCT image quality assessment and contributes to establishing a standardized framework for performance evaluation and optimization in clinical practice. It defines a methodology, based on phantoms of common use and a worldwide accepted approach, for the assessment of the PCCT scanner image quality and reports the scanner characterization for the most common scanning protocols with the ambition of setting reference values for quality assurance programs. In doing so, it expands the available literature and supports the ongoing integration of PCCT technology into clinical workflows.

## 2. Materials and Methods

### 2.1. PCCT Scanner Specification and Acquisition Protocols

Images were acquired using the clinical PCCT scanner Naeotom Alpha^®^ (Siemens Healthineers, Erlangen, Germany). The scanner is provided with two X-ray Vectron tubes and two QuantaMax photon-counting detectors (120 and 144 modules). Each tube operates at 90 kV, 120 kV, and 140 kV, with a maximum power of 120 kW. The simultaneous use of the two tubes—‘Flash’ mode—permits fast acquisitions and is mainly used in cardiological examinations. The nominal in-plane spatial resolution is 0.11 mm. Image reconstruction was performed using CT-specific iterative algorithms. The PCCT scanner was introduced with a novel iterative reconstruction algorithm, quantum iterative reconstruction (QIR). For this scanner, the available reconstruction kernels go from QIR-1 to QIR-4 [33] The Naeotom Alpha was accepted and confirmed for clinical use in our Institute in March 2024.

### 2.2. Image Acquisitions

Table 1 shows all the protocols and scanner parameters analyzed in this work. From now on we will refer to the acquired protocols as ‘H’ for head district, as ‘TA’ for the thorax/abdomen district, and as ‘IE’ for the inner ear district. Phantom tests were conducted with the Catphan600 (The Phantom Laboratory, Greenwich, NY, USA) with the purpose of task-based analysis. This phantom is commonly used for routine quality assurance (QA) and to perform acceptance tests of the CT scanner. The Catphan phantom, with its modules, assures reproducibility of the measurements, guaranteed by a suitable support and markers for the alignment with the gantry laser. Acquisitions were performed using institutional clinical CT protocols—which had been previously validated by expert radiologists—focusing on head, thorax/abdomen, and inner ear, as described in Table 1. Therefore, the selected parameters (tube voltage, exposure, and slice thickness) reflect clinically optimized settings routinely employed in patient examinations. In particular, although the inner ear protocol belongs to the head district, it features the ultra-high-resolution (UHR) acquisitions, including the possibility of reconstructing the final slice at 0.2 mm. To determine the image acquisition parameters (e.g., tube potential and exposure), we first acquired a fully automatic scan with tube current modulation. Then, the tube voltage and the exposure (mAs) automatically used by the scanner for the homogeneous uniformity Catphan were set during the measurements. Image reconstruction was performed using the default iterative algorithms for the selected acquisition protocols, as indicated in Table 1.

Regarding the delivered dose for each measurement, we previously measured the CTDI_vol_ using the standard dedicated phantoms and a 10 cm active length sensor (RaySafe X2 CT Sensor). These values were compared with those reported by the CT system, observing differences from 0.11% to 6.83%.

### 2.3. Image Quality Metrics and Analysis

The image quality analysis was performed following the American Association of Physicists in Medicine (AAPM) Task Group (TG) report 233 guidelines for testing CT systems [32]. Analysis was performed with the support of freely available software. Uniformity index (UI), integral non-uniformity (NUI), slice thickness, CNR, and SNR were calculated by employing Pylinac v. 3.33.0 [34], which is a Python-based open-access library for automated analysis in medical physics quality assurance systems. This Python library covers the most common QA procedures in medical physics, particularly focusing on radiotherapy applications. Pylinac takes as input the Catphan CT scan (in our case, we imported the Catphan600 module) in a DICOM (.dcm) format and then analyzes it automatically according to the user requests, by writing a simple script. The accuracy of CT numbers and image histograms was evaluated with ImageJ (Wayne Rasband, National Institute of Health, Bethesda, MD, USA) and analyzed with Python 3.9. Task-based metrics (e.g., target transfer function, NPS, and d′) were calculated using ImQuest (Duke University, Durham, NC, USA) software [35], as suggested by the AAPM TG-233 [32].

#### 2.3.1. Uniformity Index (UI) and Integral Non-Uniformity (IN)

UI and IN are parameters defined to compare uniformity between different scan protocols [36]. UI is defined as follows:

(1) $$100\cdot \frac{{\bar{HU}}_{\mathrm{periphery}}-{\bar{HU}}_{\mathrm{center}}}{{\bar{HU}}_{\mathrm{center}}+1000},$$

where ${\bar{HU}}_{\mathrm{periphery}}$ is the mean value of CT numbers measured in four 20 mm ROIs of 13 slices of the CTP486 module of the Catphan phantom, positioned at top, right, left, and bottom at 55 mm from the center of the phantom. ${\bar{HU}}_{\mathrm{center}}$ is the mean value of the CT numbers for a 20 mm ROI located in the center of the phantom [37], calculated for 13 slices in the uniformity module. UI is typically used to evaluate the amount of capping or cupping artifacts in CT images. A positive UI indicates an underestimation of attenuation values at the center of the phantom (cupping), while a negative UI indicates an overestimation (capping). IN is defined as

(2) $$IN=\frac{{\bar{HU}}_{\max}-{\bar{HU}}_{\min}}{{\bar{HU}}_{\max}+{\bar{HU}}_{\min}+2000},$$

where ${\bar{HU}}_{\max}$ and ${\bar{HU}}_{\min}$ are, respectively, the maximum and minimum values of the mean of CT numbers in five ROIs located at the center and lateral positions of the phantom 34. IN quantifies the overall variation of attenuation values within a homogeneous phantom region, highlighting spatial differences in the system’s response.

#### 2.3.2. Slice Thickness

The CTP404 module contains four 23° tilted wire ramps. Slice thickness is measured by determining the FWHM of these ramps in the CTP404 module. A profile of the area around each wire ramp is taken, and the FWHM is determined from the profile. Slice thickness is then obtained by multiplying by tan(23°). For each wire ramp, the corresponding slice thickness is calculated, and the final value is obtained by averaging these four measurements.

#### 2.3.3. Contrast-to-Noise Ratio (CNR) and Signal-to-Noise Ratio (SNR)

The low-contrast module (CTP515) of the Capthan was used for calculating the CNR and SNR. Contrast is calculated by considering a ROI within the low-contrast insert and a ROI in the background, using the following relationship:

(3) $$c=\frac{{\bar{HU}}_{\mathrm{insert}}-{\bar{HU}}_{\mathrm{bkg}}}{{\bar{HU}}_{\mathrm{insert}}+{\bar{HU}}_{\mathrm{bkg}}}$$

where ${\bar{HU}}_{\mathrm{insert}}$ is the mean of the insert ROI and ${\bar{HU}}_{\mathrm{bkg}}$ is the mean of the background ROI (Michelson contrast [34]). The CNR is then calculated by dividing the contrast by the noise value, estimated as the standard deviation of the background ROI (CNR=c(I)/σ(I)). The SNR is calculated by dividing the average intensity in the ROI by the standard deviation of the background. Pylinac calculates the CNR and SNR values for the 1%-contrast ROIs (and not for 0.5% and 0.3% objects). This is because the software works with a threshold, set according to the visibility of the single contrast inserts. Objects at 0.5% and 0.3% were visible for all the acquired scans. We did not perform a manual evaluation as it is not reproducible across all the acquisitions.

#### 2.3.4. Image Histograms and CT Numbers

For each CT scan, HU histograms were obtained by extracting 128 × 128-pixel ROIs in the central slice of the CTP486 module. HU medians, interquartile range (IQR), kurtosis, and skewness were calculated from the histograms. For each scan, the bin width was set to 1 HU, and the number of bins was set equal to the total HU values contained in the selected ROI. CT numbers of all the inserts located in the CTP404 module were estimated. These inserts were made of air, Polystyrene, Delrin, acrylic, Teflon, LDPE, and PMP. HU values were assessed as the mean values over a circular ROI with a diameter of 8 pixels. Besides the CT numbers values, we were interested in assessing the linearity of the HU values versus the linear attenuation coefficients calculated for the corresponding X-ray spectrum of each scan. We first estimated the half value layers (HVLs) of the X-ray spectra by performing three static repeated scans with the tube in 0° position and a calibrated dosimetry system (RaySafe X2 R/F Sensor) positioned directly on the opposite side of the gantry. HVL resulted in 6.87 mm Al at 120 kV and 7.60 mm Al at 140 kV. These values confirmed the measurements of the acceptance tests of the CT scan in our institution. The corresponding spectra were calculated via TASMICS v. 1.7 [38], by selecting aluminum as the material filtration of the tube and inserting the measured values of HVLs. We extrapolated the effective beam energy (keV), which was then used to calculate the linear attenuation coefficients from the database of the National Institute of Standards and Technology (NIST) [39]. For each scan of the phantom, we performed a linear regression between the measured HU values and the computed linear attenuation coefficients.

#### 2.3.5. Noise Texture and Noise Power Spectrum

**Figure 1:**
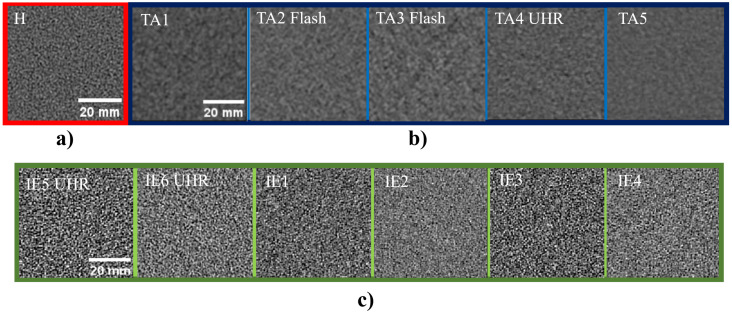
(**a**) 128 × 128 noise ROI for head protocol; (**b**) noise ROIs for all the analyzed thorax/abdominal protocols; (**c**) noise ROIs for all the analyzed inner ear protocols.

A qualitative analysis of the noise texture of the images was performed by cropping 128 × 128-pixel ROIs in the central homogeneous slice of the CTP486 module (Figure 1). Afterwards, for the calculation of the noise power spectrum (NPS), we used ImQuest noise tab. We manually positioned eight 25 mm square ROIs in all the slices included in the uniformity module. The software calculates the NPS as follows:

(4) $${\mathrm{NPS}}_{2D}\left(f_{x},f_{y}\right)=\frac{\Delta_{x}\;\Delta_{y}}{L_{x}\;L_{y}}\cdot \frac{\sum_{i=1}^{N_{\mathrm{ROI}}}{\left|{\mathrm{FFT}}_{2D}\left\{ROI_{i}\left(x,y\right)-FIT_{i}\left(x,y\right)\right\}\right|}^{2}}{N_{\mathrm{ROI}}}$$

where $\Delta_{x}$ and $\Delta_{y}$ are the pixel spacing in x and y directions, $L_{x}$ and $L_{y}$ are the ROI dimensions, ${\mathrm{ROI}}_{i}$ is the considered ROI among the 8 drawn in the slice, ${\mathrm{FIT}}_{i}$(x,y) represents the second-order polynomial fit of the ${\mathrm{ROI}}_{i}$(x,y). ${\mathrm{FFT}}_{2D}$ is the bidimensional (2D) fast Fourier transform. Once the ${\mathrm{FFT}}_{2D}$ is obtained, all radial profiles are averaged to produce NPS1D curves. When calculating the NPS, both the peak frequency ($f_{peak}$) and the average frequency ($f_{average}$) are considered for performance quantification.

#### 2.3.6. Spatial Resolution

Spatial resolution was assessed by considering a task-based metric, which is the target transfer function (TTF). In particular, we focused on three specific materials present as inserts in the phantom (CTP404 module): Teflon and Derlin, which mimic hyperdense areas (e.g., calcifications, hyperdense brain lesions), and Polystyrene, which mimics soft-tissue equivalent materials (2% contrast, which is the lowest in this module of the chosen phantom). TTF was calculated with ImQuest, by positioning 8 ROIs on the circular inserts in all the visible slices of the CTP404 module. ROIs were resized to obtain the same diameter as the Catphan inserts. The following formula explains the practical calculation of TTF:

(5) $$\mathrm{TTF}\left(f\right)=\frac{\left|\int \frac{d}{dx}\mathrm{ESF}\left(x\right)\;e^{-i2\pi fx}\;dx\right|}{\int \frac{d}{dx}\mathrm{ESF}\left(x\right)\;dx}$$

In this equation, ESF(x) is the edge spread function evaluated for each ROI. It is generated by binning and averaging pixel CT numbers as a function of radial distance. Equation (5) is the Fourier transform of the derivative of the ESF, which is the line spread function (LSF), normalized for the DC component. For TTF, $f_{50\%}$ and $f_{10\%}$ are considered as reference performance parameters.

#### 2.3.7. Detectability Index

Detectability index (d′) is a task-based detection performance metric. It can be calculated as follows [40]:

(6) $${d^{\prime }}^{2}=\frac{{\left[\;\int \int \;{\left|W_{\mathrm{task}}\left(u,v\right)\right|}^{2}\;{\mathrm{TTF}}^{2}\left(u,v\right)\;du\;dv\right]}^{2}}{\;\int \int \;{\left|W_{\mathrm{task}}\left(u,v\right)\right|}^{2}\;{\mathrm{TTF}}^{2}\left(u,v\right)\;\mathrm{NPS}\left(u,v\right)\;du\;dv}$$

In this equation, u, v represent the spatial frequencies and $W_{task}$ is the Fourier transform of the signal that has to be detected (task function). In this work, we considered a circular task function with a diameter of 5 mm and calculated d′ for each insert (contrast) of the phantom. We set a non-pre-whitening matched filter (NPW), which is an observer model that compares the image of interest to a template consisting of the expected signal via cross-correlation. This task function was chosen as it correlates well with human performance in low-contrast detectability [41].

## 3. Results

The order of the listed protocols in the following tables will be the same as in Table 1.

### 3.1. Uniformity, Slice Thickness, and Low-Contrast Detail Visibility

**Figure A1:**
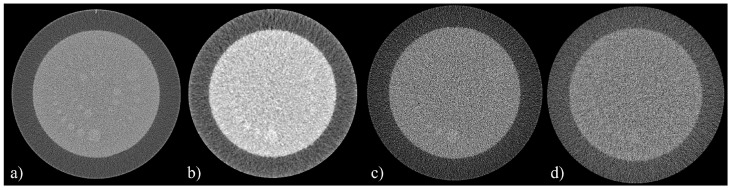
Low-contrast module of the Catphan600 phantom shown for some of the acquired districts: (**a**) Head; (**b**) Abdomen; (**c**) Inner Ear at standard mode (144 × 0.4 mm); (**d**) Inner Ear at UHR (120 × 0.2 mm). Window/Level settings were chosen to achieve the best visibility possible of the low-contrast objects.

Table 2 shows the values of uniformity index, integral non-uniformity, slice thickness, CNR, and SNR. CTDI_vol_ values were also reported for each acquisition. CNR and SNR were calculated for the largest and smallest ROIs of the 1%-contrast objects in the CTP515 module of the Catphan600. UI ranges from −0.0986 to +0.1994 for abdominal acquisitions, and from −0.0764 to +0.0578 for the inner ear protocol. Analysis of the UI and NI revealed no clear pattern indicative of capping (positive values) or cupping (negative values) artifacts, suggesting that, in this case, such artifacts are not strongly associated with IR algorithms or acquisition parameters. It is also worth noting that the uniformity and the SNR of this CT scanner are monitored quarterly as part of the medical physics quality assurance (QA) program. Slice thickness, measured with the tilted ramp method, depends on the protocol and reconstruction algorithm, but remains within tolerance limits (±0.5 mm for collimations < 1 mm). For CNR and SNR, the values tend to be smaller for IE acquisitions; as we will discuss in Section 3.3 and Section 3.5, this could be influenced by the greatest noise that characterized these acquisitions, which also affects the detectability of low-contrast details. A qualitative demonstration of the visibility of the low-contrast objects can be found in Appendix A (Figure A1). These images clearly show that, as the acquisition resolution increases, very-low-contrast objects become difficult to detect. As even the 1% contrast inserts are barely distinguishable, the CNR and SRN evaluations for the 0.5% and 0.3% contrast levels were not feasible.

### 3.2. Image Histograms

**Figure 2:**
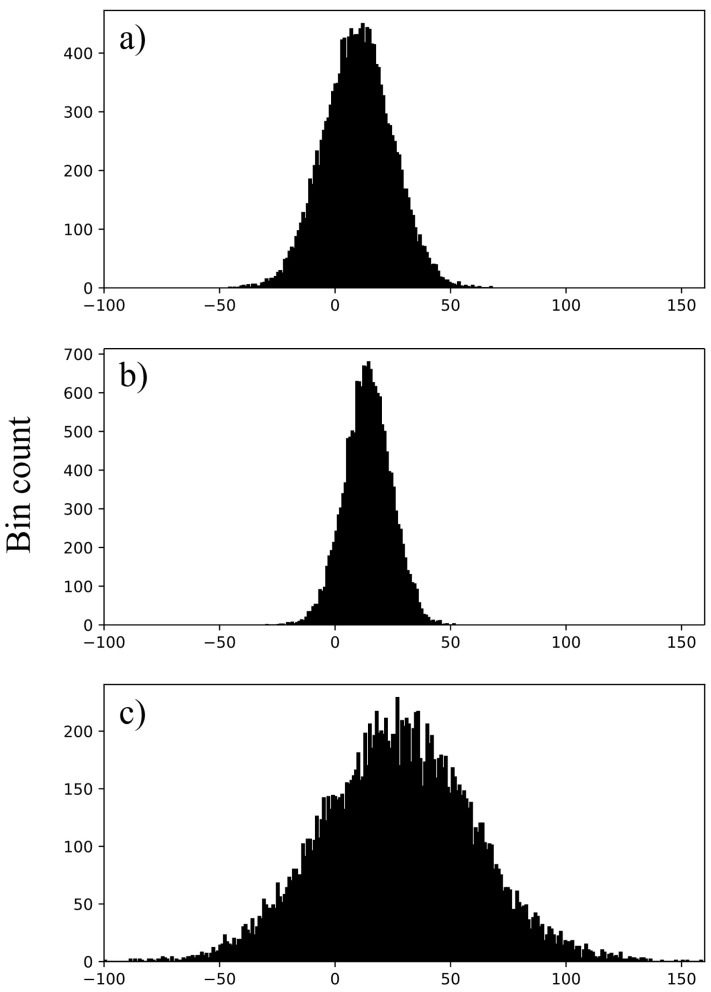
(**a**) HU distribution for head protocol; (**b**) HU distribution for abdomen protocol; (**c**) HU distribution for inner ear protocol.

The uniformity module of the Catphan600 was also analyzed through image histograms, obtained from a 200 mm × 200 mm squared ROI centered in the phantom. Table 3 collects HU medians, interquartile range (IQR), kurtosis, and skewness. The median HU values vary depending on the selected protocol, ranging from 4 HU to 14 HU for abdominal acquisitions and from 16 HU to 28 HU for ear protocols. IQR is significantly larger for ear districts compared to head or abdomen (maximum value is 70 HU). This is also illustrated in Figure 2, which shows the image histograms for head, abdomen, and ear protocols.

### 3.3. Noise Power Spectrum

**Figure 3:**
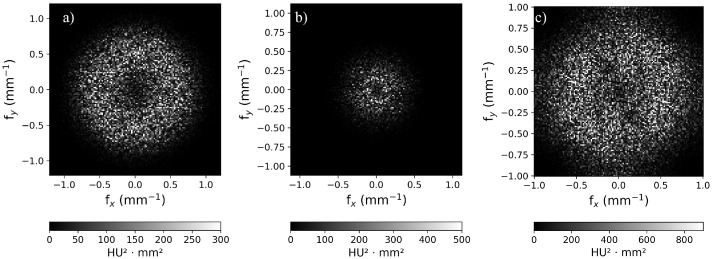
Representation of NPS2D for (**a**) head, (**b**) abdomen, and (**c**) inner ear UHR. Axis ticks refer to the spatial frequencies in x–y directions.

**Figure 4:**
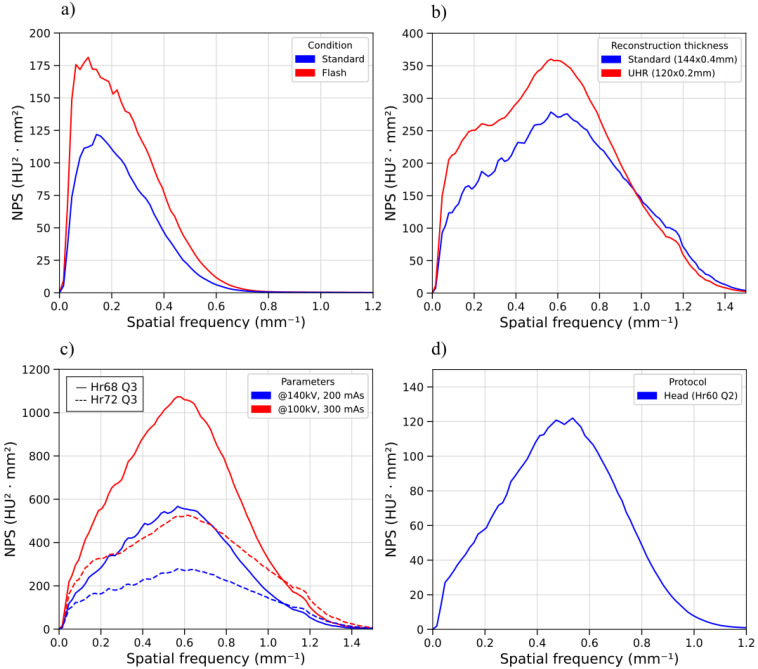
(**a**) Comparison between standard and flash abdomen protocols; (**b**) NPS curves for two acquisition collimation widths (144 × 0.4 mm and 120 × 0.2 mm) in the inner ear protocol; (**c**) comparison between two reconstruction kernels (Hr68 and Hr72) at two different CTDIvol values for inner ear; (**d**) NPS for the head protocol.

Figure 3 shows the 2D-NPS for three selected acquisitions (head, thorax/abdomen, inner ear UHR). Figure 3 reports the NPS for the selected protocols. Figure 4a compares the NPS for the standard and flash abdominal protocols modalities. Figure 4b shows the NPS curves for the inner ear acquisition protocols, comparing collimation widths of 144 × 0.4 mm and 120 × 0.2 mm (UHR). In particular, the UHR curve shows a higher peak in terms of NPS. This could be affected by the choice of reconstructing the image slice at 0.2 mm rather than 0.4 mm. Figure 4c refers to two different iterative reconstruction kernels for the inner ear protocol and two different values of CTDIvol (5.1 mGy for the red curves and 13.3 mGy for the blue curves). The kernels chosen were Hr68 (solid line), and Hr72 (dashed line), both with a level 3 of strength (Q3). Peak frequencies are similar in these cases, but Hr68 acquisitions are noisier than Hr72. Figure 4d characterizes the NPS as a standard head protocol (CTDIvol: 44.92 mGy). The differences in noise texture in the spatial domain are highlighted in Figure 1, which shows 128 × 128 ROIs cropped from the central homogeneous slice of the uniformity module of the phantom, for each acquisition protocol listed in Table 1. As shown in Figure 4b, Hr72-inner ear acquisitions at UHR and 144 × 0.4 mm exhibit a shoulder in the NPS curve at approximately 0.25 ${\mathrm{mm}}^{-1}$. This trend is particularly visible in UHR reconstructions and could probably be associated with the non-linearity of the iterative reconstruction algorithm. Table 4 collects the peak, average frequencies, and noise magnitude of NPS curves for each acquisition protocol: for ear acquisitions, f_peak_ is in the range of 0.57–0.59 ${\mathrm{mm}}^{-1}$, showing minimal variation across the parameters described in Table 1. Furthermore, Figure 4d and Table 4 show that the noise power spectrum trend differs slightly between the standard head protocol and the ear protocol, with the former exhibiting a f_peak_ of 0.50 ${\mathrm{mm}}^{-1}$ and an f_average_ of 0.49 ${\mathrm{mm}}^{-1}$. The noise magnitude (HU) values—obtained as the square root of the area under the NPS2D curves—clearly show the dependence of noise on the dose level, the chosen reconstruction algorithm (for example, comparing IE1 with IE3, and IE2 with IE4), and total collimation width (IE3 vs. IE5, IE4 vs. IE6).

### 3.4. Target Transfer Function

**Figure 5:**
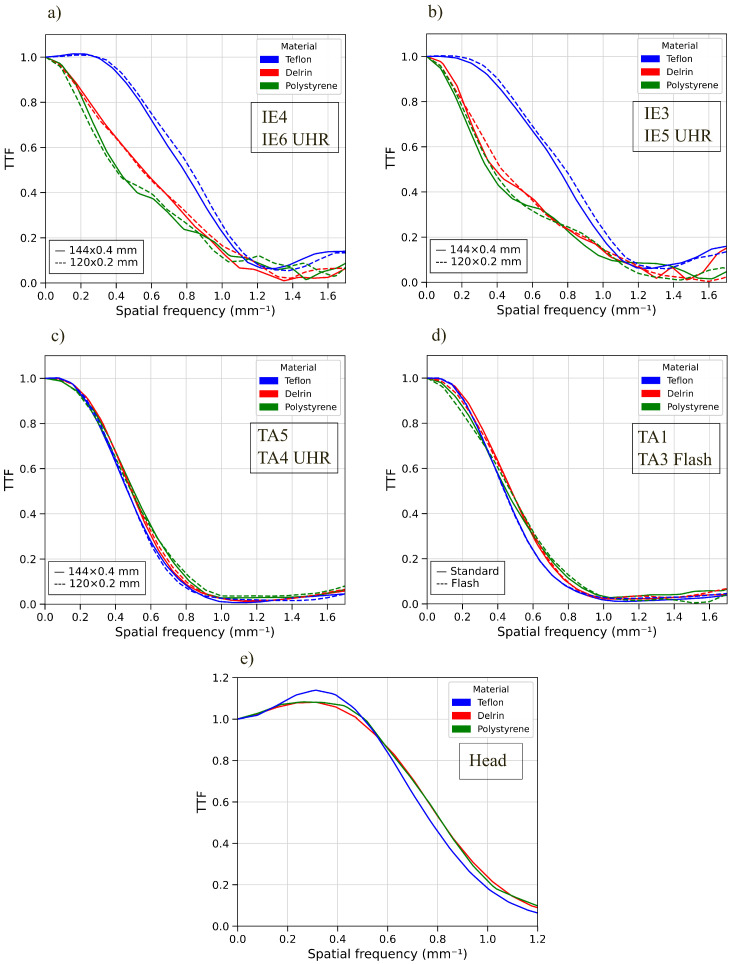
(**a**) TTF curves for inner ear at 140 kV, 200 mAs comparing 144 × 0.4 mm and 120 × 0.2 mm collimation widths; (**b**) TTF curves for inner ear at 100 kV, 300 mAs comparing 144 × 0.4 mm and 120 × 0.2 mm collimation widths; (**c**) TTF curves for thorax/abdominal protocol; (**d**) TTF curves for thorax/abdominal protocol comparing standard and flash acquisitions; (**e**) TTF curves for the head protocol.

We calculated the TTF curves for each protocol, considering the inserts in the CTP404 module of the phantom. Figure 5 presents the TTF curves for three materials: Delrin, Teflon, and Polystyrene. Figure 5a,b show the TTF curves for two different CTDIvol values, corresponding to acquisition parameters of 140 kV coupled with 200 mAs and 100 kV coupled with 300 mAs, respectively. Figure 5c represents the abdominal protocol acquired at 140 kV and 100 mAs, while Figure 5d compares standard and flash abdominal protocols. The trend of TTF for these inserts varies noticeably across protocols. Of particular interest are the Teflon curves for ear acquisitions: focusing on a specific image, such as the one acquired at 140 kV and 200 mAs with Hr72 reconstruction kernel, the $f_{50\%}$ for Teflon is 0.82 ${\mathrm{mm}}^{-1}$, compared to 0.53 ${\mathrm{mm}}^{-1}$ for Delrin. A higher $f_{10\%}$ is observed for the Teflon insert.

### 3.5. Detectability Index

**Figure A2:**
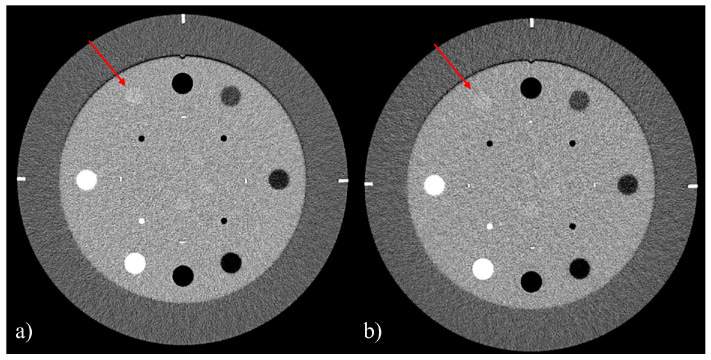
CTP404 module shown for the Inner Ear acquisitions: (**a**) standard (144 × 0.4 mm), (**b**) UHR (120 × 0.2 mm) acquisitions. The red arrow shows the polystyrene insert used for TTF calculation.

The d′ values for each insert of the CTP404 modules are reported in Table 5. d′ increases with the contrast of the material against the background, as higher-contrast inserts (e.g., Teflon) are more easily detected than lower-contrast ones (e.g., acrylic). In UHR reconstructions, d′ values are lower compared to acquisitions with larger collimation widths (e.g., 140 × 0.4 mm). This can be related to the increased noise of the image (Figure 3) which affects the low-contrast detectability in the phantom. For example, comparing abdominal protocols evaluated at 0.4 mm and 0.2 mm reconstruction thicknesses at the same CTDI_vol_ (11.6 mGy) d′ values range from 3.96 for acrylic to 138.82 for air. A qualitative demonstration of this behavior is reported in the Appendix A (Figure A2), where a comparison of the standard and UHR mode for the module CTP404 is shown. The image shows the degradation of low-contrast visibility for the Polystyrene object when switching from standard to UHR modality.

### 3.6. CT Numbers

**Figure 6:**
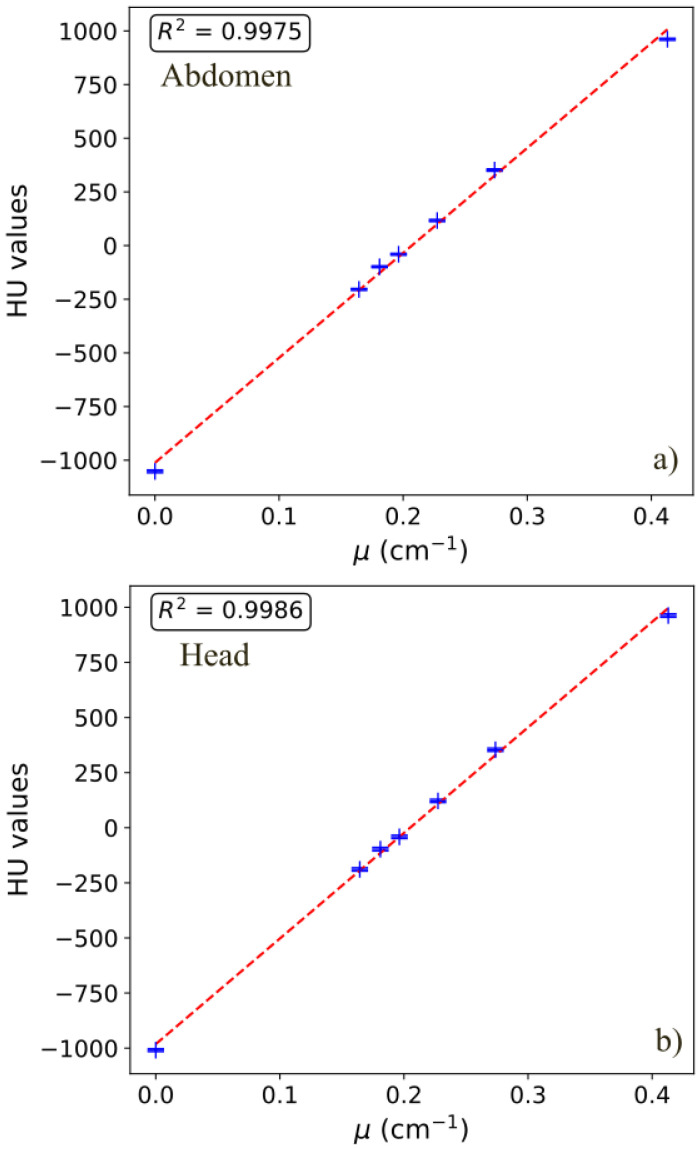
Linearity of the HUs vs. linear attenuation coefficients computed for the specific spectrum, for abdominal (**a**) and head (**b**) protocols.

Table 6 reports the HU values for the inserts of the Catphan600 phantom. R^2^ values, reflecting the linearity of the HUs with respect to the linear attenuation coefficients computed for the specific spectra, are also shown. Standard deviations range from a minimum of 1.2 HU to a maximum of 14.7 HU. Figure 6 shows two linearity plots (head and abdomen) as an example (120 kV and 140 kV spectra). Table 6 highlights that different acquisition protocols and parameters affect the CT numbers for the same materials: for example, for the Polystyrene insert, CT number varies from −51.8 HU (flash abdomen) to −14.8 HU (UHR inner ear Hr72).

## 4. Discussion

This work performed a thorough technical characterization of a clinical PCCT scanner, providing baseline parameters in terms of both basic image quality metrics and task-based metrics, which are more indicative of the real diagnostic purposes. Head, thorax/abdomen, and inner ear protocols were investigated in this phantom study. The measurements with the commercial phantom assure high reproducibility and, eventually, allow comparison with other commercial scanners (e.g., EID-CTs) or intervariability studies with other institutes. To our knowledge, this is the first comprehensive work about this novel clinical scanner, providing essential technical information for clinical practice and the definition of standardized quality assurance (QA) programs [32]. The novelty of this work, together with the current lack of research on this specific scanner, makes it difficult to provide a direct comparison of our findings with previously published data. Some works [28,30,31] have recently demonstrated the capabilities of a PCCT scanner in reducing the delivered dose by preserving image quality. Greffier et al. (2025) [41] demonstrated that, when reconstructing the standard and UHR images at the same slice thickness (0.4 mm), the noise magnitude from the UHR mode is reduced with respect to standard-acquisition images. Basic image quality metrics such as uniformity, SNR, CNR, and slice thickness are regularly monitored in our medical physics QA program. The observed CNR and SNR results for low-contrast details, especially for IE acquisitions, align with the findings from NPS and d′. NPS reveals a greater noise magnitude in the case of UHR (120 × 0.2 mm) with respect to 144 × 0.4 mm acquisitions, as quantified by the square root of the area under the NPS2D curves, as shown in Figure 4b,c. As an example, at a CTDI_vol_ of about 5 mGy (acquisition parameters of 100 kV and 300 mAs), the noise magnitude for the standard mode was 41.0 HU, while for the UHR mode it was 42.3 HU. Furthermore, as shown in Figure 4c, the gap in the noise magnitude decreases when increasing the reconstruction kernel. This is also confirmed by Martinez et al. (2023) [31] findings. When reconstructing at the same slice thickness (e.g., 0.4 mm for both standard and UHR modes), we observed an increase in the noise magnitude of the standard acquisition (41.0 HU), rather than the UHR mode (33.6 HU), as confirmed by the evaluations described in [31]. The values of d′ for the low-contrast insert (acrylics) show differences of 42% between 0.4 mm slice thickness and 0.2 mm slice thickness at about 13 mGy, and differences of 13.9% for a CTDI of about 5 mGy. A similar behavior (37.3%) is observed when comparing UHR and standard thorax/abdomen images at 11.6 mGy. Increased noise led to lower values for CNR and SNR, impairing low-contrast lesion detectability. The degradation of d′ in the UHR protocol quantitatively demonstrates that, despite enhanced TTF, the increased noise amplitude dominates under low-contrast conditions. This evaluation is limited to the 1% contrast objects—as already clarified in Section 2—because the Pylinac software performed the calculation only for the most visible inserts. To provide a reproducible and repeatable evaluation, we did not manually calculate the CNR and SNR for the 0.5% and 0.3% objects, as their visibility was very poor (see also Figure A1 in the Appendix A). The noise pattern itself varies significantly among different acquisition protocols and IR algorithms, as highlighted qualitatively in Figure 4 and quantitatively in Table 4, where IE reconstructions exhibit higher frequency noise patterns than thorax/abdomen. This may also explain the larger interquartile ranges (IQRs) observed in CT number distributions. For each protocol, linear correlation between the measured HU values in the CTP404 module and the calculated μ (cm^−1^) at the corresponding spectra was found. We also noticed that the protocol/district, rather than the IR algorithm [21], affects the mean values of CT numbers (TA shows a mean value of −1034.4 HU for air, while IE shows −977.95 HU (Table 6)). CTDIvol only affects the standard deviation of measurements. These values are also confirmed by image histogram parameters (Figure 2). Regarding the TTF values, as described in Section 2.3, we focused on three materials of interest for clinical tasks, which are Teflon, Delrin, and Polystyrene. For thorax/abdomen protocols, TTF curves largely overlap, as shown in Figure 5. On the other hand, for the IE protocol, Teflon curves, for both CTDIvol values investigated in this study, exhibit higher f_50%_ than Delrin and Polystyrene. We can attribute this result to the fact that the Teflon insert has an HU range between 941 HU and 1060 HU that is very comparable to the bone. Supporting this, noise texture and NPS analyses show that IE images appear ‘crisper’ compared to other protocols. The TTF curves varying with the contrast insertsis attributable to the non-linearity of the iterative reconstruction algorithms, which affect the relationship between signal, noise, spatial resolution, and contrast, depending on both kernel characteristics and strengths. The non-linearity of the reconstruction algorithms has an impact also on noise, in particular on noise magnitude, as there is not a clear dependence—from the data presented in this work—between the input dose level and the output noise magnitude, according to the algorithm chosen (see Table 4). Masturzo et al. (2025) [27] performed a thorough analysis of the dependence of noise on the type of reconstruction algorithm (whether filtered backprojection or iterative) and the corresponding strength. Previous studies have demonstrated, from both a qualitative and quantitative approach, the suitability of UHR acquisitions for temporal bone imaging [42,43]. There are no significant differences between the TTF curves of standard and UHR images for any material in this study. Despite the improvement in the spatial resolution due to the use of PCDs, it is worth noting that there are still intrinsic physical mechanisms that may limit this aspect, such as the charge-sharing and the k-escape, that spread the signal beyond the interaction location within the detector. These effects may be reduced by means of a larger pixel size or with the support of a suitable algorithm, also exploiting the most advanced deep learning methodologies [44] or a convolutional neural network [45]. To better contextualize the results presented in this work, it is important to highlight how specific physical mechanisms intrinsic to photon-counting detectors influence the observed image quality metrics. First, the limited difference observed between the standard and UHR TTF curves (<5–8%) can be explained by the interplay between reduced pixel size and charge-transport phenomena in CdTe detectors. In particular, charge-sharing and K-escape events introduce an effective broadening of the point-spread function that partially counteracts the theoretical resolution gain expected from the 0.2 mm slice thickness [46].

The main limitation of this study resides in the use of a single phantom, which does not take into account all the morphologies of the patients undergoing CT examinations. Despite the high reproducibility offered by the used phantom (e.g., same orientation and positioning for each acquisition, possibility of quantitative analysis for each module), anatomy-simulating phantoms could be more suitable to evaluate the anatomical background and anisotropic structures. We focused our analysis on the default reconstruction kernels for each district to reproduce, in the best way possible, the clinical conditions of acquisitions. Further investigations could be made on other reconstruction kernels and on the virtual monoenergetic images (VMIs), as shown in [30] for the monenergetic images at 70 and 73 keV. Few works have performed an image quality comparison with an EID-CT [31,41] using phantoms. The comparisons available in the literature mainly regard the image quality evaluated directly on clinical images to assess the impact of using one scanner or the other for clinical outcomes. However, the acquisition conditions and the reconstruction parameters always differ between the two scanners [47].

## 5. Conclusions

This study provides the first phantom-based characterization and baseline parameters of a clinical whole-body PCCT scanner. Both standard and task-based metrics were calculated and described in this paper, considering standard (144 × 0.4 mm) and UHR (120 × 0.2 mm) collimation widths. We found that the outcomes in terms of image quality strongly depend on the chosen acquisition protocol, as shown by the HU medians, noise texture, and TTF. Furthermore, we observed that, for this kind of scanner, the total collimation width affects the noise magnitude and, consequently, the detectability index related to low-contrast inserts. This was particularly evident for the ear protocol that allows UHR acquisitions. On the contrary, spatial resolution associated with a high-contrast clinical task (Teflon insert) increased for the same protocol.

## Figures and Tables

**Table 1:** List of the acquisition parameters set in PCCT for the acquisition of the Catphan600 phantom.

District (Abbreviation)	Protocol	kV	mAs	Slice Thickness (mm)	Collimation Width	Axial Pixel Size (mm)	Recon. Algorithm	Pitch
Head (H)	H	120	266	1.0	144 × 0.4	0.4135 × 0.4135	Hr60 Q2	0.35
Thorax/Abdomen (TA)	TA1	120	30	1.0	120 × 0.2	0.4271 × 0.4271	Br40 Q4	0.85
	TA2 Flash	140	30	1.0	144 × 0.4	0.4271 × 0.4271	Br40 Q4	3.2
	TA3 Flash	120	30	1.0	144 × 0.4	0.4271 × 0.4271	Br40 Q4	3.2
	TA4 UHR	140	100	0.2	120 × 0.2	0.4365 × 0.4365	Br40 Q4	0.85
	TA5	140	100	0.4	144 × 0.4	0.4365 × 0.4365	Br40 Q4	0.85
Inner Ear (IE)	IE1	100	300	0.4	144 × 0.4	0.4199 × 0.4199	Hr68 Q3	0.55
	IE2	140	200	0.4	144 × 0.4	0.4199 × 0.4199	Hr68 Q3	0.55
	IE3	100	300	0.4	144 × 0.4	0.4199 × 0.4199	Hr72 Q3	0.55
	IE4	140	200	0.4	144 × 0.4	0.4199 × 0.4199	Hr72 Q3	0.55
	IE5 UHR	100	300	0.2	120 × 0.2	0.4199 × 0.4199	Hr72 Q3	0.85
	IE6 UHR	140	200	0.2	120 × 0.2	0.4199 × 0.4199	Hr72 Q3	0.85

**Table 2:** UI, IN, slice thickness, CNR, and SNR for all the acquisition protocols listed in Table 1. CTDI_vol_ for each protocol is shown in the last column.

Protocol	UI	IN	Slice Thickness (mm)	CNR (1%, 15 mm)	SNR (1%, 15 mm)	CNR (1%, 5 mm)	SNR (1%, 5 mm)	CTDI_vol_ (mGy)
H	0.002	0.297	0.907	0.008	5.144	0.006	3.746	44.9
TA1	0.199	0.001	1.293	0.018	7.367	0.009	10.762	2.4
TA2 Flash	−0.099	0.001	1.208	0.014	10.714	0.012	9.060	3.4
TA3 Flash	−0.099	0.001	1.010	0.013	8.822	0.023	9.809	2.4
TA4 UHR	0.076	0.062	0.646	0.014	8.113	0.009	6.394	11.6
TA5	0.101	0.065	0.794	0.015	10.216	−0.015	9.423	11.7
IE1	−0.076	0.066	0.690	0.002	1.227	$4\times 10^{-5}$	1.089	5.1
IE2	0.024	0.024	0.791	0.002	2.366	$4\times 10^{-4}$	1.861	13.3
IE3	0.059	0.055	0.754	0.002	1.717	$2\times 10^{-4}$	1.451	5.1
IE4	−0.049	0.052	0.617	0.002	2.952	−0.001	2.647	13.3
IE5 UHR	−0.032	0.074	0.498	0.001	1.736	0.002	1.932	5.1
IE6 UHR	−0.053	0.063	0.462	0.002	2.563	−0.001	2.348	13.5

**Table 3:** Median of HU values, IQR, kurtosis, and skewness obtained from image histograms in the uniformity module of the Catphan phantom.

Protocol	Median (HU)	IQR (HU)	Kurtosis	Skewness
H	10	20	0.009	−0.025
TA1	4	10	−0.035	−0.007
TA2 Flash	13	11	0.126	0.022
TA3 Flash	14	12	0.067	−0.035
TA4 UHR	10	11	0.008	0.037
TA5	10	7	0.021	−0.062
IE1	18	69	0.034	0.012
IE2	27	49	−0.001	0.070
IE3	17	54	0.070	−0.021
IE4	28	39	0.123	−0.007
IE5 UHR	16	54	0.111	0.005
IE6 UHR	28	42	0.056	0.003

**Table 4:** Collection of f_peak_, f_average_, and noise magnitude (HU) for each acquired protocol.

Protocol	f_peak_ (mm^−1^)	f_average_ (mm^−1^)	Noise Magnitude (HU)
H	0.50	0.49	23.4
TA1	0.16	0.24	8.1
TA2 Flash	0.13	0.24	8.3
TA3 Flash	0.09	0.23	10.1
TA4 UHR	0.22	0.26	8.5
TA5	0.19	0.26	5.7
IE1	0.57	0.58	52.5
IE2	0.57	0.58	38.1
IE3	0.59	0.62	41.0
IE4	0.59	0.62	30.8
IE5 UHR	0.58	0.56	42.3
IE6 UHR	0.58	0.57	32.5

**Table 5:** d′ values for some of the protocols described in this work. Material contrasts with respect to the background are reported in brackets.

Protocol	Parameters	Acrylic (30)	Polystyrene (−120)	Air (−1065)	LDPE (−160)	PMP (−250)	Teflon (800)	Delrin (240)
**Head (H)**	Hr60 (44.32 mGy), 1 mm	11.38	56.25	467.54	80.92	121.37	372.59	106.23
**Inner Ear (IE)**	UHR Hr72 (5.12 mGy)	3.38	15.21	144.45	22.41	32.16	108.24	29.74
	UHR Hr72 (13.5 mGy)	6.13	35.36	318.26	51.16	76.17	230.28	66.19
	Hr72 (5.04 mGy), 0.4 mm	3.93	29.07	265.10	42.45	62.62	200.78	53.22
	Hr72 (13.3 mGy), 0.4 mm	10.59	44.06	412.75	65.58	100.39	294.66	88.62
**Thorax/Abdomen (TA)**	Br40 (2.36 mGy), 1 mm	4.74	35.29	258.53	51.39	72.00	205.56	65.80
	Br40 (3.44 mGy) Flash, 1 mm	5.06	41.12	322.64	60.77	88.84	261.07	73.76
	Br40 (2.36 mGy) Flash, 1 mm	4.25	28.24	220.34	41.14	59.75	163.62	52.30
	UHR Br40 (11.6 mGy)	6.66	39.90	318.37	57.59	84.57	248.73	76.52
	Br40 (11.6 mGy), 0.4 mm	10.62	59.29	457.19	83.97	124.45	360.21	108.81

**Table 6:** HU values for each insert for acquisition protocols investigated in this work. The last column reports R^2^ values obtained from the linear regression against the computed linear attenuation coefficients.

Protocol	Polystyrene	Delrin	Teflon	Air	Acrylic	PMP	LDPE	R^2^
H	−40.6	350.8	955.5	−1008.0	119.7	−188.4	−97.6	0.9986
TA1	−51.0	353.3	981.6	−1032.5	116.4	−201.9	−110.5	0.9988
TA2 Flash	−51.8	363.0	979.7	−1040.1	111.5	−193.3	−110.2	0.9980
TA3 Flash	−36.6	353.8	964.5	−1055.5	122.1	−198.8	−97.0	0.9975
TA4 UHR	−43.3	359.6	980.5	−1033.9	120.8	−194.4	−102.8	0.9986
TA5	−43.4	360.6	982.0	−1034.4	122.1	−195.1	−103.0	0.9984
IE1	−27.6	349.8	919.0	−985.1	126.6	−173.9	−82.6	0.9972
IE2	−15.8	358.3	900.9	−983.8	134.3	−163.6	−71.5	0.9951
IE3	−32.2	341.9	909.0	−978.7	120.0	−174.7	−85.6	0.9972
IE4	−15.8	355.1	899.3	−982.4	133.4	−161.2	−70.1	0.9953
IE5 UHR	−29.2	343.8	910.1	−967.7	121.9	−169.3	−83.9	0.9974
IE6 UHR	−14.8	350.3	891.1	−970.0	134.2	−159.9	−69.5	0.9951

## Data Availability

The original contributions presented in this study are included in the article. Further inquiries can be directed to the corresponding author.

## Appendix A

A visual representation of the low-contrast objects from the CTP515 module of the phantom is shown in this Section (Figure A1). It is also reported a comparison between the CTP404 modules for the Inner Ear at a CTDI_vol_ of 13 mGy between standard and UHR acquisitions at the same reconstruction kernel of Hr72 (Figure A2).
